# Miniaturized and Wireless Optical Neurotransmitter Sensor for Real-Time Monitoring of Dopamine in the Brain

**DOI:** 10.3390/s16111894

**Published:** 2016-11-10

**Authors:** Min H. Kim, Hargsoon Yoon, Sang H. Choi, Fei Zhao, Jongsung Kim, Kyo D. Song, Uhn Lee

**Affiliations:** 1 Center for Materials Research, Norfolk State University, Norfolk, VA 23504, USA; m.kim@spartans.nsu.edu (M.H.K.); ksong@nsu.edu (K.D.S.); 2 Neural Engineering and Nano Electronics Laboratory, Department of Engineering, Norfolk State University, Norfolk, VA 23504, USA; 3 NASA Langley Research Center, Hampton, VA 23681, USA; sang.h.choi@nasa.gov; 4 Department of Chemical and Biological Engineering, Gachon University, Seongnam 13120, Korea; zhaofei0911@gmail.com (F.Z.); jongkim@gachon.ac.kr (J.K.); 5 Department of Neurosurgery, Gachon University of Medicine and Science, Incheon 21936, Korea; uhn@gachon.ac.kr

**Keywords:** microspectrometer, fluorescence, dopamine, optical sensing, wireless

## Abstract

Real-time monitoring of extracellular neurotransmitter concentration offers great benefits for diagnosis and treatment of neurological disorders and diseases. This paper presents the study design and results of a miniaturized and wireless optical neurotransmitter sensor (MWONS) for real-time monitoring of brain dopamine concentration. MWONS is based on fluorescent sensing principles and comprises a microspectrometer unit, a microcontroller for data acquisition, and a Bluetooth wireless network for real-time monitoring. MWONS has a custom-designed application software that controls the operation parameters for excitation light sources, data acquisition, and signal processing. MWONS successfully demonstrated a measurement capability with a limit of detection down to a 100 nanomole dopamine concentration, and high selectivity to ascorbic acid (90:1) and uric acid (36:1).

## 1. Introduction

Real-time sensing of dopamine (3,4-dihydroxyphenethylamine) (DA) activity in the brain is critically important to understand neural behavior and to develop therapeutic intervention technologies for neurological disorders and diseases. DA concentration in the extracellular space of brain tissues varies depending on both tonic and phasic dopamine release mechanisms [1]. Tonic dopamine release results from spontaneous single spikes in DA neurons, with a concentration of a few tens of nanomoles (e.g., 10–20 nM within the striatal region [2]), depending on the number of DA neurons responding during spontaneous spike activities. Conversely, phasic release of DA reflects the burst activity of firing neurons and is regulated by glutamergic excitatory synaptic drive from other brain areas, including the pedunculopontine tegmentum (PPTg) and the subthalamic nucleus (SN) [2,3,4,5]. The burst spike firing can increase the local dopamine concentration to the millimolar range.

There have been many different types of dopamine sensing technology using electrical and optical sensing principles. Among these multiple neural sensing methods, electrical sensing of neurotransmitters was carried out with amperometry and voltammetry methods. For dopamine, fast-scan cyclic voltammetry is a well-known technique with high accuracy. Application of a high scan rate allows reversible dopamine to be oxidized on an electrode and reduced back in a short time period, before significant molecular diffusion on the electrode occurs. Therefore, dopamine can be detected by redox reaction on the electrode, even in the presence of interference by irreversible species. Since the temporal resolution of fast scan cyclic voltammetry (FSCV) is limited to measuring sub-second fluctuation of dopamine transients [6,7], fixed potential methods, such as single potential amperometry or bipotential amperometry, are used for measuring oxidation or oxidation/reduction currents. Fixed potential amperometry can measure sub-millisecond dopamine activity [8,9].

A complementary neural sensing method is optical neural sensing technology [10,11,12,13,14,15,16,17,18]. Optical sensing of dopamine was performed by surface-enhanced Raman spectroscopy (SERS) [19,20,21,22,23,24,25] and fluorescence spectroscopy in vitro and in vivo. For in vivo optical dopamine sensing, excitation is delivered to a sensing point in the brain and the responsive optical spectrum from dopamine is measured by a spectrometer. In recent studies, nanostructures and materials have been integrated for enhanced sensitivity and selectivity [17,19,25]. With strongly amplified electromagnetic interaction and charge transfers within nanoscale dimensions and material structures, the optical sensing of dopamine was possible at micro/nanomole scales, and even at a single molecular level. In addition, optical sensing using photoluminescence phenomena can provide the capability of measuring the basal level change (tonic) of neurotransmitter concentration, compared to the conventional fast-scan cyclic voltammetry that can only measure short term differences.

For optical dopamine sensing in the brain, optical fibers are used for transferring probe lights and responding signals. Collected optical spectra of signals are analyzed by a spectrometer to identify the neurotransmitter and measure amounts and changes with time. Optical neurotransmitter sensing has been performed under tethered connection of optical fibers and electrical cables between the animal and data acquisition units, including spectrometers and system electronics. However, the tethered connection limits animal behaviors and significantly restricts experimental studies. To solve this issue, wireless sensing technology was developed and applied for many experiments, ranging from the use of small insects to non-human primates and human subjects [26].

The main objective of this research is to develop a wireless optical dopamine sensor that can measure phasic DA release and uptake in the extracellular space from a few micromoles to millimoles of concentration. This kind of device can be integrated with deep brain stimulation (DBS) systems for adaptive neuromodulation. DBS offers a new alternative to treat neurological disorders, such as Parkinson disease (PD), depression, dystonia, and Tourette syndrome [27]. DBS involves the implantation of electrodes precisely at neuro-anatomical targets related to the disease. However, current DBS technology is hindered by several limitations, such as lack of automatic parameter adjustment and the inability to continuously adapt to clinical fluctuations, which are typical phenomena in advanced Parkinson’s disease. Therefore, the adaptive DBS, in conjunction with a miniaturized and wireless optical neurotransmitter sensor (MWONS), would be greatly beneficial, providing the capability of automatic control according to the changes in a patient’s symptoms and condition.

We previously presented a preliminary design of an optical dopamine sensing system, measured its outputs, and characterized its response [28,29]. Here we introduce a miniaturized wireless dopamine sensor as an integral part of a micro-spectrometer, a data acquisition unit, and a wireless data transfer module. This new sensor has successfully demonstrated capabilities of dopamine sensing with high performance using an integrated optical sensing probe.

## 2. Materials and Methods

**Figure 1:**
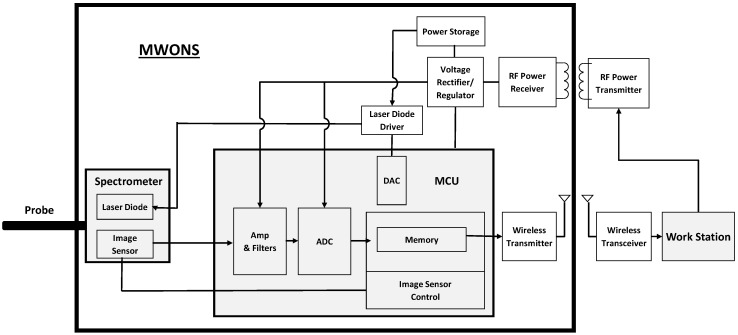
Schematic diagram of the wireless optical sensing system.

The miniaturized and wireless optical neurotransmitter sensing system implements fluorescence microscopy. For in vivo sensing in the brain, an optical fiber delivers a probe beam for singlet excitations and receives emission spectra from singlet transitions. Fluorescence as the emission spectra corresponding to the changes in neurotransmitter concentration is measured by MWONS. MWONS consists of three major parts: a fluorescence sensing probe, a micro-spectrometer unit, and a system electronics module with a microcontroller unit (MCU) that controls the spectrometer and performs data acquisition and analysis, power management, and wireless communication. Figure 1 shows the schematic diagram of the overall sensing structure, including the remote workstation, which can be a laptop computer or other portable electronic device.

### 2.1. Micro-Spectrometer and Sensing System Electronics

#### 2.1.1. Micro-Spectrometer Design

**Figure 2:**
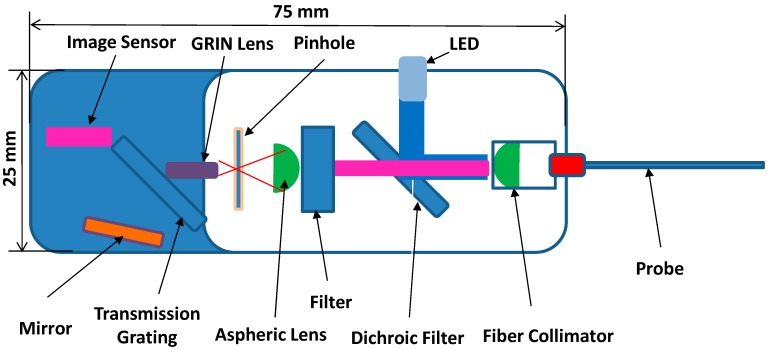
Schematic diagram of a micro-spectrometer.

For biomedical research and clinical applications, miniaturization of a spectrometer is critically important. The micro-spectrometer is one of the most important components in optical sensing that transduce optical responses into electrical signals. Extensive research has been performed to enhance optical resolution and sensing speed, while reducing the physical size [30,31,32]. For biomedical or behavioral animal research, a miniaturized spectrometer is essential for implantation or loading when using an animal model. The micro-spectrometer was developed using the Fresnel diffraction principle [30,33,34] for medical applications. A schematic diagram of a currently used micro-spectrometer for fluorescence dopamine sensing is shown in Figure 2.

In this design, the light for photo-excitation of the target comes from a light emitting diode (LED) that is collimated, filtered, and diffracted through optical components assembled in a small package with a size of 25 mm (W) × 75 mm (L) × 15 mm (H). The collimation of the emission spectra from the target object into the optical probe and filter units was obtained by aspheric lenses. The focal length and numerical aperture (NA) of the aspheric lens are selected for optimum efficiency in a limited volume of the system package. For sensing the fluorescence from the emissive target, which is different from the wavelength of excitation, a dichroic mirror and a long-pass filter are adapted in this design. Based on excitation and emission spectra measurements of quantum dots, a dichroic filter with a cutoff wavelength of 506 nm, a reflection wavelength of 440–500 nm, and a transmission wavelength of 513–730 nm were used to selectively filter the excitation wavelength of 465 nm. A long-pass filter with a cut-off wavelength of 575 nm sharply transmits fluorescence signals (605 nm) from the sensing head of the probe. As a compact form of a collimating unit, a gradient index rod (GRIN) lens is placed between a pin hole and a diffraction grating. For sensing the diffracted signals, a linear CMOS image sensor (ELIS-1024, Dynamax Imaging LLC, Canandaiqua, NY, USA) with an array of photodiode pixels is used. The sensitivity of the photo-diode pixels from a 400 nm to 900 nm wavelength is mostly linear.

#### 2.1.2. Sensing Electronics Design

As shown in Figure 1, which describes the overall system configuration of the optical dopamine sensing system, the core electronic system is comprised of programmable system-on-chip version-5 (PSoC5, Cypress, San Jose, CA, USA) microcontroller embedding analog and digital peripheral functions, including analog-digital converters (ADCs), digital-analog converters (DACs), amplifiers, and analog and digital input/output ports in a chip. Analog output signals from the image sensor in the spectrometer are captured and converted into digital signals by the ADCs. In this design, operating clock signals for the ADCs that come from the MCU are synchronized with the image sensor operation. In the sensor application software, control commands are sent to the control unit so that control parameters of the image sensor and ADCs can be adjusted.

The ADCs perform with the sampling frequency of 2 MHz and 16-bit resolution. All captured 16-bit ADC outputs are stored in a memory via direct memory access functions. The memory has an 8-bit structure while converted data are 16-bit forms. The 16-bit data are separated into one high 8-bit part and the other low 8-bit part and stored in the memory respectively. When it is transmitted by the wireless transmitter, 128 low memory bytes and 128 high memory bytes are sent and reconstructed at the workstation. For wireless data communication, a Bluetooth module (RN-42, Microchip Technology Inc., Chandler, AZ, USA) is chosen due to its simplicity and low power consumption. Universal asynchronous receiver/transmitter (UART) protocol with 115,200 bps speed is used for the communication interface between the microcontroller and the workstation.

For control of injection light intensity for photo-excitation and exposure time, the FL500 light emitting diode (LED) driver chip is employed. This chip can control the operating current of an LED with constant current in the range from 0 mA to 250 mA. In the system, the MCU can control the optical output of the LED by applying input voltages from 0 V to 2 V using DACs. The developed neural recording system requires 2.5 V for ADC operation and 3.3 V for the rest of modules. The power supply for 2.5 V and 3.3 V is regulated by a low dropout regulator (LDO, TPS61200, Texas Instruments, Dallas, TX, USA), supplying stable power to each component. In this system, an AC-DC converter and DC charging module is also integrated, which will be implemented for future integration of this sensing unit with the DBS module and wireless power transfer [29,35].

#### 2.1.3. 3D Printing Fabrication and Assembly

**Figure 3:**
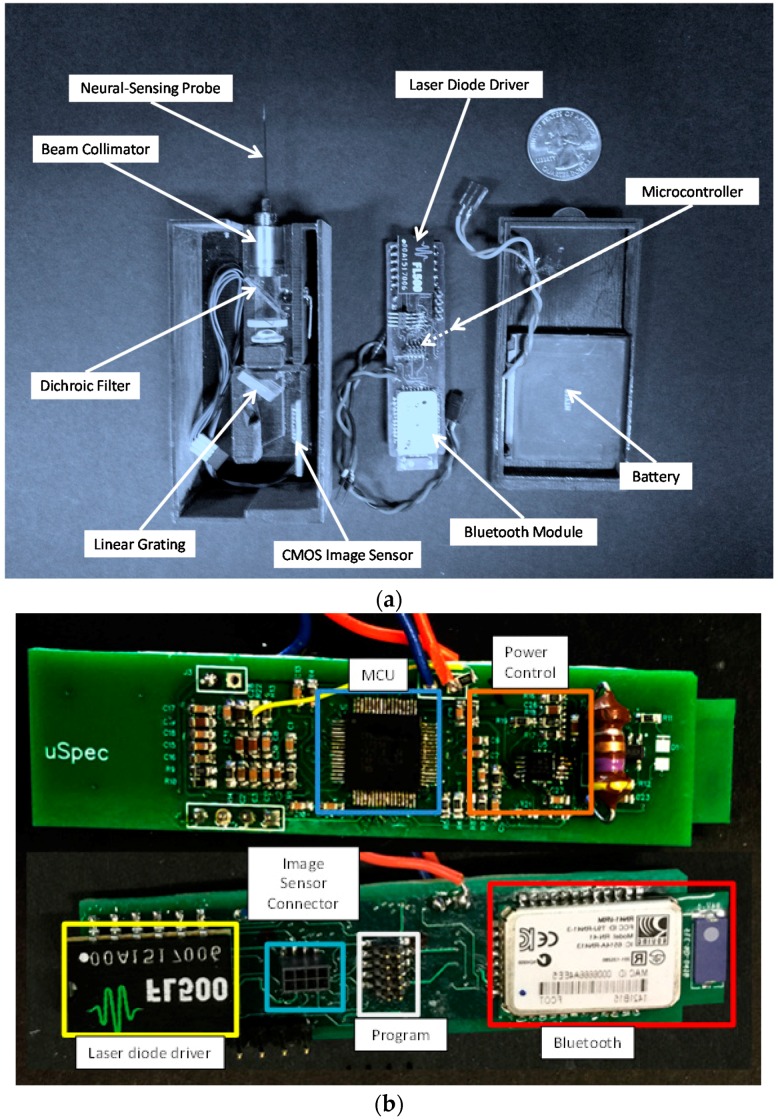
(**a**) Image of sensing components and assembly in a 3D printed package and (**b**) the front and backside images of a printed circuit board with assembled electrical parts.

3D printing technology was used to fabricate the frame of the optical and electrical components, with the advantages of rapid and low cost prototyping, as shown in Figure 3. For the micro-spectrometer, several spectrometer designs were prototyped by a 3D printer and tested for an optimum structural and functional design to enhance alignment and beam path. Alignment of each optical part was examined by measuring the signal intensity and resolution of signal spectrum and adjusted to reach an optimum value of sensing resolution and sensitivity. The base material for 3D printing is a photosensitive polymer with the elastic modulus of 1 GPa and the material density of 1.13 g/cm^3^, which shows sufficient mechanical strength for reliable operation and light weight, appropriate for future experiments in animal models. For system electronics, four-layer printed circuit boards were used to integrate functional modules in a compact layout. The size of the optical sensing system is 43 mm × 85 mm × 29 mm, after the entire module assembly, including a rechargeable battery.

### 2.2. Fluorescence Dopamine Sensing Probe

#### 2.2.1. Materials

Hydrofluoric acid (HF, 49%) was purchased from J.T. Baker (Center Valley, PA, USA). 3-aminopropyl trimethoxysilane (APTMS, 98%), dopamine hydrochloride, and sodium hydroxide (NaOH, 99.5%) were purchased from Sigma-Aldrich (St. Louis, MO, USA). Optical fibers, with a silica core diameter of 600 ± 10 mm and a clad diameter of 660 ± 10 mm, were obtained from Polymicro Technologies (Phoenix, AZ, USA). Quantum dots (QDs) in phosphate-buffered saline (PBS) (EM: 605 nm) were purchased from ThermoFisher Scientific (Waltham, MA, USA). All reagents were used without any further purification.

#### 2.2.2. Design and Fabrication

**Figure 4:**
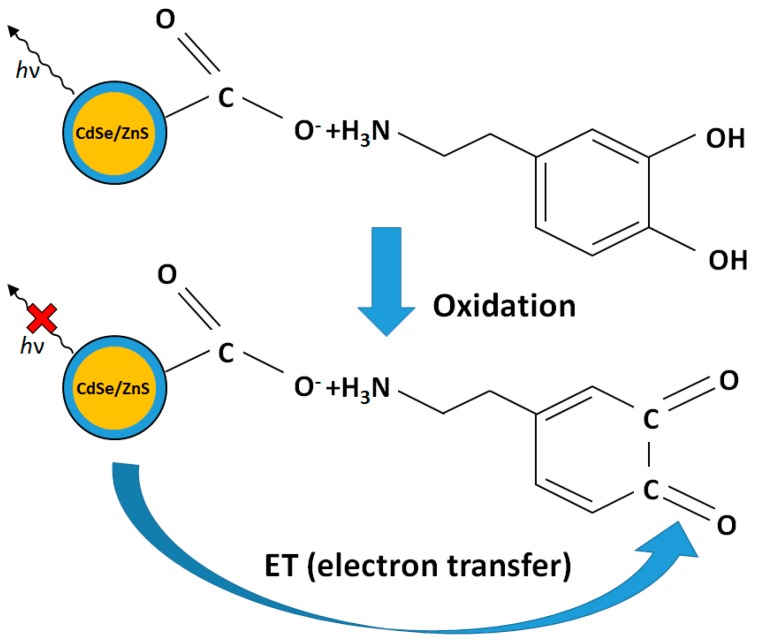
Schematic diagram of luminescence change (quenching) the QDs by electron transfer.

The fluorescence neurochemical method is advantageous because of the relatively strong signal intensity and high signal-to-noise ratio compared to other forms of optical sensing. In this research, CdSe/ZnS QDs were used at the tip of an optical fiber to detect dopamine by measuring signal intensity changes by fluorescence quenching and recovering. QDs have peculiar optical properties due to their confined excitations in three dimensions and have been used as novel fluorescent tools for sensing and imaging applications [36,37]. Compared with traditional organic fluorophores, QDs have many superior properties, such as high stability, against photo bleaching, high quantum yield, tenability, and extendable emission spectra, and narrow emission spectral width. Interaction of the carboxyl-QDs with dopamine results in the formation of QDs/dopamine, which are further oxidized to the o-quinone residues by oxygen. The latter products are expected to act as quenchers to turn off the fluorescence of QDs due to electron transfer. This mechanism provides a path for the optical detection of dopamine, as shown in Figure 4.

**Figure 5:**
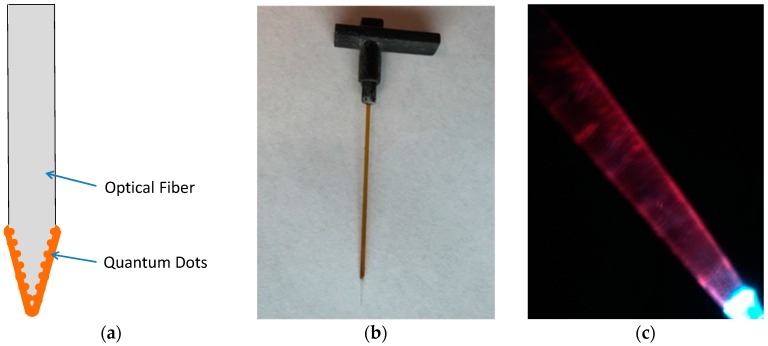
(**a**) Schematic diagram of the optical probe, and images of (**b**) optical probe with a connector and (**c**) probe tip with QDs photo luminescence excited with a 465 nm light.

For optical sensing probes, optical fibers with 600 µm/650 µm (core/clad) diameter were used. An optical fiber tip was dipped in sulfuric acid at 100 °C to remove the polyimide cladding. After cleaning with ddH_2_O, the fiber was immersed in gradually-injected hydrofluoric acid through a syringe pump and allowed to air-dry. The surface of the tapered optical fiber tip was silanized with an amine functional group by using 3-aminopropyltriethoxysilane (APTES). The fiber tip was then incubated overnight in a mixture of 400 mL of 100/100 mM EDC/ NHS (prepared in 0.05 M borate buffer, pH 7.4) and 10 μL (8 mM) of QDs, and kept in darkness under ambient temperature. The fabricated fibers were cleaned by PBS buffer solution to remove loosely-bound QDs. The size of the fiber tip with QDs can be controlled, and 2–3 mm lengths of QD tips were made and tested in this research. The schematic diagram of the optical probe with QDs and images are shown in Figure 5.

## 3. Experiments and Results

### 3.1. Characterization of Optical Sensing Probes

**Figure 6:**
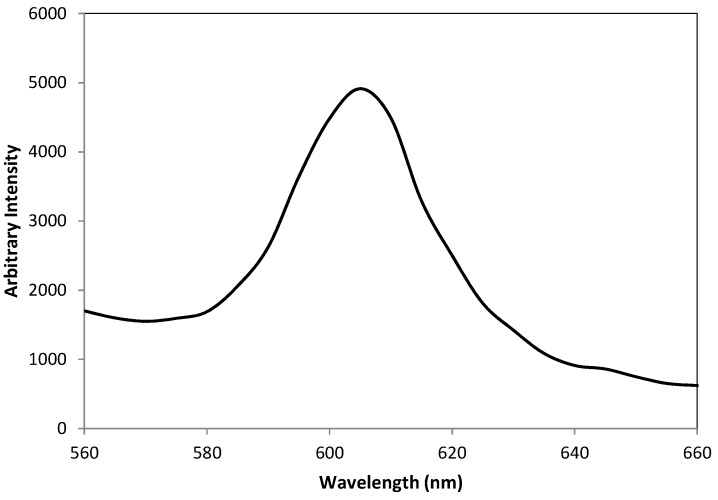
Photoluminescence spectrum for a 470 nm wavelength excitation.

Fluorescence spectra of QDs were characterized using a QuantaMaster™ 50 PTI spectrofluorometer (Photon Technology International, Birmingham, NJ, USA) to find the optimum wavelength for the excitation source and emission signals. The optimum excitation wavelength was checked by measuring the emission spectra from the optical probe by scanning the excitation wavelengths from 450 to 500 nm. From repeated measurements, it was found that 465 nm excitation provide the maximum output of 605 nm emission from the QDs at the tip of probe. An example of a photoluminescence spectrum for a 470 nm wavelength excitation is shown in Figure 6.

**Figure 7:**
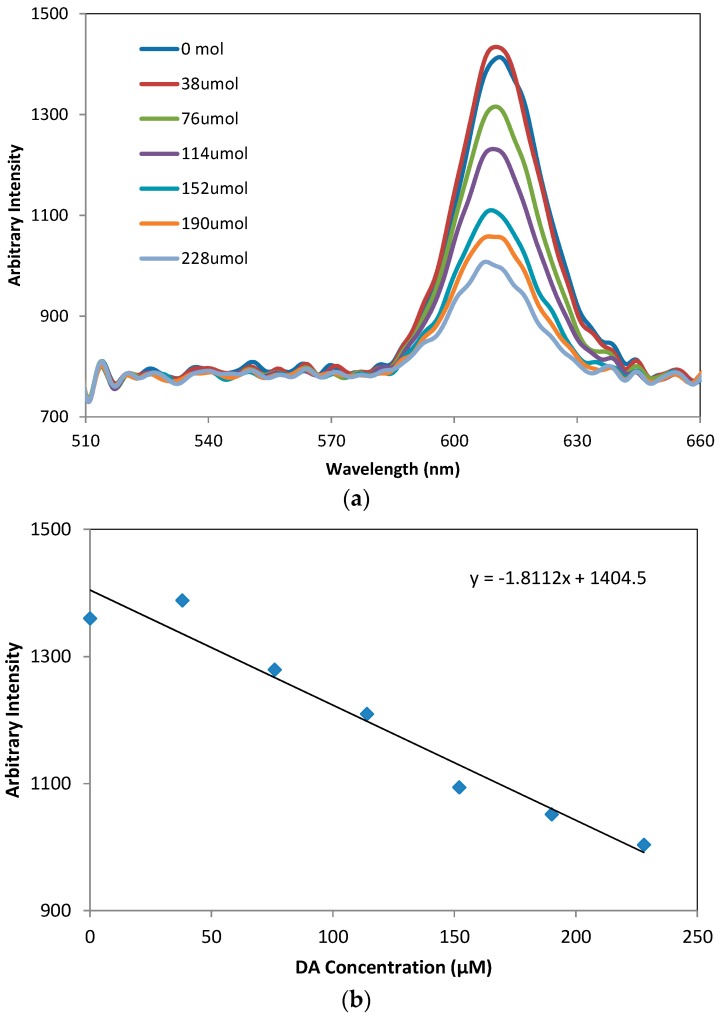
(**a**) Photo-luminescence spectra of CdSe/ZnS QDs at different concentrations of DA from 0–228 µM and (**b**) the relation between the luminescence spectra and DA concentration measured by a commercial spectrometer (USB 4000).

In fluorescence dopamine sensing with QDs, the interaction of carboxyl-QDs with dopamine results in formation of QDs/dopamine complexes that are further oxidized to o-quinone residues by oxygen. In this research, the latter products were designed to act as quenchers to turn off the fluorescence of QDs due to electron transfer. This mechanism provides a path for the optical detection of dopamine by measuring the intensity of fluorescence. Figure 7a shows the change of photo-luminescence spectra of CdSe/ZnS QDs at different concentrations of DA from 0–228 µM using a commercial spectrometer (USB 4000, Ocean Optics, Dunedin, FL, USA). The fluorescence intensity is linearly correlated to DA concentration and decreases with the increase of DA concentration, as shown in Figure 7b.

### 3.2. Characterization of the Optical Dopamine Sensor

**Figure 8:**
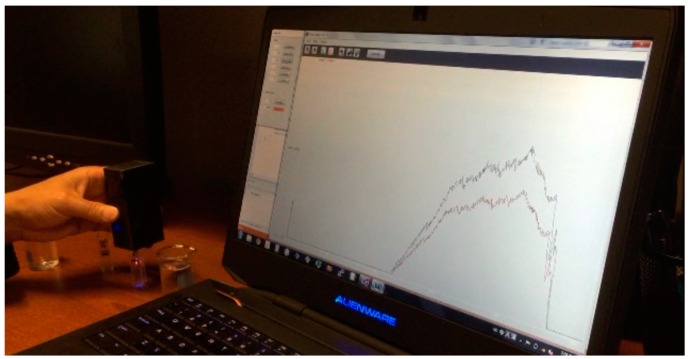
Custom-designed graphical user interface for the sensor control and sensing data display.

The function of the micro-spectrometer module and sensing electronics was tested in benchtop experiments. The fluorescence quenching signals from the optical probe with QDs were measured by dipping the probe in various concentrations of dopamine in the phosphate-buffered saline solution. When the measurements were carried out, sensing signals from the CMOS image sensor were adjusted for a proper level of signal-to-noise ratios by amplifying signals, adjusting offsets, and controlling the duration of data acquisition and intensity of excitation lights. A custom designed graphical user interface (GUI) programmed with the C# language in the workstation included these control functions and displayed the sensing signals received via a Bluetooth wireless connection, accessible by a laptop computer in real-time, as shown in Figure 8.

**Figure 9:**
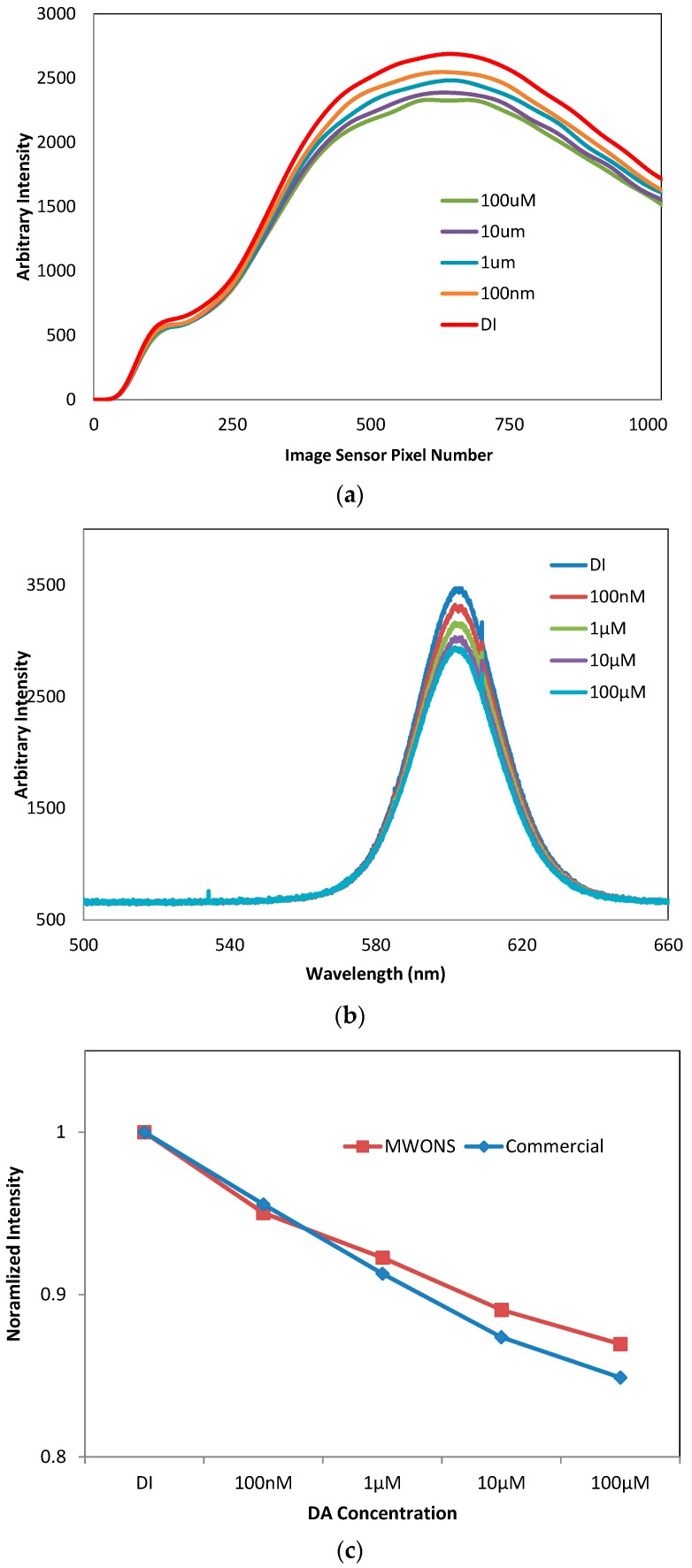
DA sensing sensitivity measured by (**a**) MWONS and (**b**) a commercial micro-spectrometer; and (**c**) comparison of sensitivity with DA concentration.

While changing dopamine concentration, the voltage signals from the optical sensing probe were measured by the image sensor reflecting the emission light from the sensing probe. This experiment was performed in a Faraday cage with a light shielding curtain to minimize interference. In Figure 9a, the sensing response from the fluorescent sensing probe was clearly detected by MWONS, with the limit of detection (LOD) down to 100 nM. This sensitivity is sufficient to cover the physiological range of extracellular dopamine concentration released by phasic neuronal activity, which is also the target of dopamine sensing for adaptive DBS. The sensitivity of our wireless dopamine sensor was compared to the sensing performance obtained by a commercially available micro-spectrometer (Figure 9b), utilizing external light source, filter block, and the USB wire connection for experiments. The comparison between the two sensing units, shown in Figure 9c, demonstrates that this newly-developed stand-alone optical dopamine sensor has high sensitivity to detect dopamine with the advantages of real-time monitoring and display via a wireless connection, and miniaturization appropriate for future application in human and animal models.

**Figure 10:**
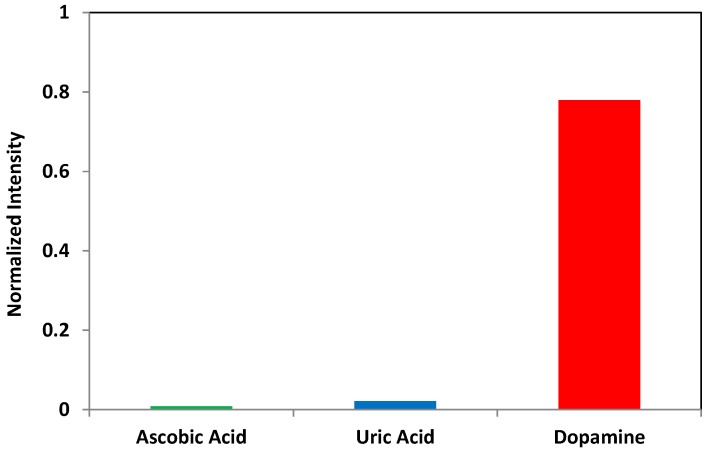
DA sensing selectivity to other biological substances, ascorbic acid (AA) and uric acid (UA).

The sensing selectivity of dopamine in the presence of other biological substances, such as ascorbic acid (AA) and uric acid (UA), was also evaluated. The signal intensity in PBS and 1 mM concentration of AA, UA, and DA was measured, respectively, using the same probe, with the ratio of signal change for each experiment calculated as the normalized values in the graph. According to the relevant literature [38,39], AA concentration is around 1–2 mM in the extracellular medium, while intracellular neuronal concentrations are much higher, reaching up to 10 mM. DA selectivity is 90:1 to AA and 36:1 to UA, as shown in Figure 10, which is high enough to sense dopamine in the normal metabolic conditions of human and animal models. This result is also similar to previous research reported by Tashkhourian et al. [40]. When electron charge transfer occurs from the conduction band (CB) of QDs to DA or AA, enolate intermediate ions are formed. Since the phenoxide and ascorbate ions are more stabilized forms, DA and AA lose a hydrogen ion. The electron on the oxygen atom is delocalized around the ring and one of the lone pairs on the oxygen atom overlaps with the delocalized electrons on the benzene and ribose rings. For ascorbic acid selectivity, it is assumed that the fluorescence of QDs was quenched more in the presence of DA than AA, because of the more resonant structure of the dopamine enolate ion than that of the ascorbate enolate ion.

When we tested optical probes with QDs, we observed a noticeable variation in sensing performance, showing a decrease of fluorescence signal intensity with time. We assumed the leaching of QDs during operation could be the main reason and the amount of QDs immobilized on the fiber tip may decrease with time. The leaching of QDs not only caused issues of sensing performance variation with time, but also may be toxic to the cells and tissues. For in vivo use of dopamine sensing, the sensor should be biocompatible to the host’s brain tissue, and stable over long periods of operating time. At the current stage of research, the toxicity of QDs may limit the use of the sensing probes in the brain. The toxicity of QDs mostly depends on physicochemical properties, such as the size, surface chemical stabilities, and mechanical stabilities [41,42]. Coating with a thin polymer layer of fluoropolymer or conduction polymer is suggested to protect the QDs from leaching while maintaining the sensing performance. The investigation of sensor surface modification with additional polymer layers remains as a further study.

## 4. Conclusions

A miniaturized and wireless optical neurotransmitter sensor (MWONS) was developed for real-time monitoring of extracellular dopamine concentration. MWONS is based on fluorescence sensing principles and comprises an optical sensing probe, a micro-spectrometer, and system electronics, including a micro-spectrometer, a data acquisition unit, a power management module, an LED driver, and a Bluetooth wireless transceiver. A custom-designed application software controls the optical excitation parameters, sensing conditions, data acquisition, and signal processing. MWONS successfully demonstrated wireless sensing performance with a 100 nM dopamine detection limit and selectivity to ascorbic acid and uric acid on the order of 90:1 and 36:1, respectively. MWONS, the first stand-alone optical dopamine sensor, to our best knowledge, has several advantages of low cost, real-time sensing via a wireless connection, and the small size for future application in the body.
